# Antibiotic susceptibility patterns among respiratory isolates of Gram-negative bacilli in a Turkish university hospital

**DOI:** 10.1186/1471-2180-4-32

**Published:** 2004-08-22

**Authors:** Ugur Gonlugur, Mustafa Zahir Bakici, Ibrahim Akkurt, Tanseli Efeoglu

**Affiliations:** 1 Department of Chest Diseases, Cumhuriyet University Medical School, 58140, Sivas, Turkey; 2 Department of Microbiology, Cumhuriyet University Medical School, 58140, Sivas, Turkey

## Abstract

**Background:**

Gram-negative bacteria cause most nosocomial respiratory infections. At the University of Cumhuriyet, we examined 328 respiratory isolates of Enterobacteriaceae and *Acinetobacter baumanii *organisms in Sivas, Turkey over 3 years. We used disk diffusion or standardized microdilution to test the isolates against 18 antibiotics.

**Results:**

We cultured organisms from sputum (54%), tracheal aspirate (25%), and bronchial lavage fluid (21%). The most common organisms were *Klebsiella *spp (35%), *A. baumanii *(27%), and *Escherichia coli *(15%). Imipenem was the most active agent, inhibiting 90% of Enterobacteriaceae and *A. baumanii *organisms. We considered approximately 12% of *Klebsiella pneumoniae *and 21% of *E. coli *isolates to be possible producers of extended-spectrum beta-lactamase. *K. pneumoniae *isolates of the extended-spectrum beta-lactamase phenotype were more resistant to imipenem, ciprofloxacin, and tetracycline in our study than they are in other regions of the world.

**Conclusions:**

Our results suggest that imipenem resistance in our region is growing.

## Background

Nosocomial bacterial pneumonia is frequently polymicrobial, with gram-negative bacilli predominating [1]. Because delays in antimicrobial treatment can lead to adverse outcomes, the choice of empirical therapy is vital. Many effective antimicrobial agents are available, but the treatment of nosocomial pneumonia remains challenging. We recently reported the antibiotic-resistance patterns of respiratory isolates of *Pseudomonas aeruginosa *in our region [2]. The current study investigates the distribution and drug resistance of other gram-negative bacteria in the respiratory secretions of hospitalized patients.

## Results

Table I and Table II present the antibiotic susceptibility patterns of our isolates. The most common organisms were *Klebsiella *spp (35%), *A. baumanii *(27%), and *E. coli *(15%). We also isolated rare organisms such as *Stenotrophomonas maltophilia, Burkholderia *spp, and *Hafnia alvei*. All studied Enterobacteriaceae (except *Enterobacter *spp) were far more susceptible to ticarcillin-clavulanate than to ticarcillin alone, which suggests that the primary mechanism of resistance in these organisms is β-lactamase production.

*K. pneumoniae *accounted for 79% of *Klebsiella *isolates. *Klebsiella *spp were generally more susceptible to the tested antimicrobials than were *Enterobacter *spp, *Serratia *spp*, *or *E. coli*. The overall resistance rates to the third-generation cephalosporins (cefotaxime, ceftazidime, and ceftriaxone) were as follows: *Klebsiella *spp, 10%–19%; *Serratia *spp, 16%–33%; and *Enterobacter *spp, 22%–45%. *Serratia *spp were less resistant to third-generation cephalosporins than *Enterobacter *spp. *E. coli *isolates resistant to piperacillin, gentamicin, and the fluoroquinolones accounted for only 4% of all E. coli isolates. Imipenem was the most active agent against our isolates.

After imipenem, ciprofloxacin, and the aminoglycosides, tetracycline was the most active agent against *A. baumanii*. Tobramycin was more effective against *A. baumanii *than against Enterobacteriaceae. Tobramycin and imipenem were the most active agents against both gentamicin- and ciprofloxacin-resistant *A. baumanii *(Table III).

We observed the ESBL phenotype in 10 *E. coli *isolates (20.8%) and 11 *K. pneumoniae *isolates (12.2%). All *K. pneumoniae *and *E. coli *isolates with the ESBL phenotype were resistant to tetracycline. Regarding *K. pneumoniae *isolates, 2 were susceptible to tobramycin, 3 to gentamicin, and 4 to ciprofloxacin, but 8 were susceptible to amikacin and imipenem. Regarding *E. coli *isolates, 4 were susceptible to tobramycin, 7 to gentamicin, 5 to ciprofloxacin, and 8 to amikacin, but all were susceptible to imipenem.

## Discussion

Contrary to the findings of the Turkish antimicrobial resistance study group [3], our *Klebsiella *isolates were more susceptible to third-generation cephalosporins (42.6% vs. 81.6% for ceftazidime; 65.8% vs. 85.7% for cefotaxime), aztreonam (44.0% vs. 65.6%), and ticarcillin-clavulanate (37.0% vs. 60.3%). *Klebsiella *spp were 81.6% susceptible to ceftazidime in our study; these rates are 96.6% in North America [4], 86.7% in China [5], 80.5% in Korea [6], 69.4% in Latin America [7], and 51.9% in India [8].

Although the isolation of *Acinetobacter *spp in respiratory specimens may reflect colonization and not necessarily infection [9], the most common site of nosocomial *Acinetobacter *infection is the lower respiratory tract, especially in mechanically ventilated patients [10]. *Acinetobacter *spp were the second most frequent gram-negative bacilli isolated from patients with pneumonia in Latin America [7]. In our survey, all compounds tested showed decreased activity among the *A. baumanii *isolates. Susceptibility to imipenem was >95% in Canada [11], India [8], and China [5]; susceptibility was 80.5% in our study and 55.5% in another study from Turkey [12]. The high prevalence of respiratory tract infections due to multiresistant *A. baumanii *will stimulate the use of carbapenems and possibly increase carbapenem resistance in our region.

Only 75% of our *E. coli *isolates were susceptible to ciprofloxacin. However, this rate was greater in Europe (95.2%) [13], North America (93.3%) [4], and Latin America (93.9%) [7]. *E. coli's *susceptibility to ceftazidime was >95% in Europe [13], North America [4], China [5], and Korea [6] but only 84.8% in Latin America [7], 69.6% in our study, and 42.1% in India [8]. Imipenem, the most active compound, inhibited 97.8% of our *E. coli *isolates. Conversely, *E. coli *strains were not resistant to imipenem in Europe [13], Latin America [7], India [8], China [5], or Korea [6].

*Enterobacter *spp showed high rates of resistance to broad-spectrum penicillins with or without β-lactamase inhibitors (41.7% resistance to ticarcillin-clavulanate) and third-generation cephalosporins (45.2% resistance to ceftazidime). The high rates of ceftazidime resistance among *Enterobacter *spp suggests a high prevalence of stably derepressed AmpC cephalosporinase-producing strains. Interestingly, resistance to third-generation cephalosporins, aztreonam, and ticarcillin-clavulanate was higher in our study (28.1%–48.0%) than with the Turkish antimicrobial resistance study group (13.3%–38.3%) [3]. In their study, no *Enterobacter *or *Serratia *isolates were resistant to imipenem. In our study, however, the rates of susceptibility to imipenem were 86.2% for *Enterobacter *spp and 76.5% for *Serratia *spp. Imipenem susceptibility for these two species was >95% in other parts of the world [7-10,13]. Moreover, *Serratia *spp were at least 95% susceptible to ceftazidime in the United States [14], Canada [11], India [8], China [5], and Korea [6].

From 1997 to 1999, ESBL detection rates in *K. pneumoniae *isolates were 45.4% in Latin America, 24.6% in the Western Pacific, 22.6% in Europe, 7.6% in the United States, and 4.9% in Canada [15]; this rate was 12.2% in our survey, but the other study from Turkey reported a rate of 60.5% [3]. During this same period, ESBL detection rates in *E. coli *isolates were 8.5% in Latin America, 7.9% in the Western Pacific, 5.3% in Europe, 4.2% in Canada, and 3.3% in the United States [15]; this rate was 20.8% in our study. The presence of the ESBL phenotype in *E. coli *isolates decreased susceptibility to the aminoglycosides, tetracycline, and ciprofloxacin but not imipenem, suggesting the presence of other resistance genes in ESBL-encoding plasmids. Despite the high percentage of ESBL production in *E. coli *isolates, antibiotics remained reasonably effective with these isolates. Imipenem was active against all ESBL-producing *E. coli *isolates. *E. coli *remained 30.0% resistant to gentamicin; this resistance rate was 75.9% in the Western Pacific, 57.8% in Latin America, 25.7% in Europe, and 21.1% in the United States [15].

Only 0.5%–0.7% of ESBL-producing *K. pneumoniae *isolates were resistant to imipenem in the United States, Latin America, the Western Pacific region, and Canada [15]. Our rate (27.2%) was very high in comparison. This finding may be due to our low number of isolates or our lack of a confirmation test for the ESBL phenotype. Resistance to tetracycline among ESBL-producing *K. pneumoniae *strains was 61.1% in Canada, 55.1% in the Western Pacific, 52.0% in Latin America, 49.5% in Europe, and 44.4% in the United States [15], but this rate was 100% in our study. Resistance to ciprofloxacin among ESBL-producing *K. pneumoniae *strains was 44.2% in the Western Pacific, 34.6% in the United States, 24.3% in Europe, 23.1% in Latin America, 22.2% in Canada, and 63.6% in our study.

We found only one imipenem-resistant *E. coli *isolate. It was resistant to ampicillin, ticarcillin, and piperacillin but susceptible to ceftazidime, ceftriaxone, and aztreonam. This profile suggests an oxacillinase with carbapenemase properties. This finding is interesting because class D enzymes have been found only in *Acinetobacter *spp [16]. Two imipenem-resistant *Klebsiella *spp were resistant to all β-lactams, including aztreonam. These species were probably expressed a metallo-β-lactamase with additional mechanisms (efflux, cephalosporinase hyperproduction) [16].

The absence of a confirmation test for the ESBL phenotype limits the impact of our results. On the other hand, it is known that supplemented media (blood) can alter the zone diameters for several agents and bacterial species. Despite these limitations, our data can be used for local therapeutic choices.

## Conclusions

We previously presented the antibiotic susceptibility patterns of 249 respiratory isolates of *P. aeruginosa *during the same period [2]. When combined with our current data, these results show that, in our region, ceftazidime can still be used for managing respiratory infections due to gram-negative aerobic bacteria in combination with aminoglycosides. It appears that increasing imipenem resistance may cause serious therapeutic problems in future.

## Methods

We collected our data from 01/01/1999 to 01/01/2002 at the microbiology laboratory of the University of Cumhuriyet. We processed the data to eliminate duplicate registrations. We excluded any isolates collected within 7 days when they came from the same specimen source of the same patient. We initially identified the isolates using such routine methods as colonial/microscopic morphology and enzymatic characteristics. We confirmed species identification with API-bioMerieux products. We retrospectively analyzed antibiotic susceptibility patterns in 238 respiratory isolates of Enterobacteriaceae members and 90 respiratory isolates of *A. baumanii*. We accepted all consecutive isolates because we did not attempt to distinguish actual pathogens from colonizing strains. Specimen types consisted of sputum (54.2%), transtracheal/endotracheal aspirates (24.6%), and bronchial lavage fluid (21.2%). We cultured sputum samples that showed no oral contamination in the presence of sputum purulence or a suspected lower respiratory infection.

We confirmed susceptibility to 18 antimicrobial agents using disk diffusion according to the National Committee for Clinical Laboratory Standards (NCCLS) guidelines [17], except insofar as we supplemented Mueller-Hinton agar with 5% defibrinated blood. We aerobically incubated the inoculated plates at 35°C and evaluated them after 24 h. For quality control of the disk diffusion tests, we used *E. coli *ATCC 25922 and *Staphylococcus aureus *ATCC 25923 strains. The disks (Oxoid) contained the following antimicrobials: ampicillin (10 μg), ampicillin/sulbactam (20 μg), piperacillin (100 μg), aztreonam (30 μg), cefazolin (30 μg), cefuroxime (30 μg), cefotaxime (30 μg), ceftriaxone (30 μg), ceftazidime (30 μg), amikacin (30 μg), gentamicin (10 μg), tobramycin (10 μg), ciprofloxacin (5 μg), imipenem (10 μg), tetracycline (30 μg), and cotrimoxazole (25 μg). Until November 1999, our microbiology laboratory based susceptibility rates on disk zone sizes; thereafter, we used a coordinating laboratory to determine the minimal inhibitory concentrations (MICs) of these 18 antimicrobial agents, accomplished with a standardized microdilution technique (Sceptor System, Becton Dickinson Microbiology System). We used this system to determine MICs for all strains. We used the NCCLS criteria to identify possible extended-spectrum β-lactamase (ESBL)-producing strains of *Klebsiella *spp and *E. coli *when MICs were increased (2 mg/mL) with ceftazidime and/or ceftriaxone and/or aztreonam [18], but we lacked a test to confirm the ESBL phenotype.

We classified our results into two categories. We labeled strains deemed susceptible by the disk diffusion method or microdilution technique as *susceptible*. We labeled all resistant and intermediate isolates as *resistant*. We divided the number of resistant isolates by the total number of isolates that had undergone susceptibility testing.

## Authors' contributions

UG had primary responsibility for study design, collection of data, and writing the manuscript. IA, MZB, TE had intellectual contribution as well as the writing of manuscript. All authors read and approved the final manuscript.

## Figures and Tables

**Table 1:** Susceptibility rates (percentages) of Enterobacteriaceae and *Acinetobacter baumanii *between January 1999 and November 1999 (Disk diffusion method).

Antibiotics	Klebsiella spp (n: 28)	E.coli (n: 18)	Proteus spp. (n: 10)	Enterobacter spp (n: 10)	A. baumanii (n: 7)	Serratia spp. (n: 6)
Ampicillin	-	33.3	-	-	-	-
Amoxicillin/clav.	7.1	33.3	-	-	-	-
Aztreonam	42.9	44.4	50.0	60.0	-	33.3
Piperacillin	32.1	27.8	50.0	30.0	-	33.3
Cefazolin	64.3	66.7	50.0	10.0	14.3	-
Cefuroxime	46.4	66.7	40.0	40.0	-	-
Cefotaxime	67.9	72.2	90.0	100.0	-	66.7
Ceftazidime	71.4	72.2	70.0	90.0	14.3	50.0
Ceftriaxone	71.4	88.9	90.0	90.0	-	50.0
Amikacin	92.9	100.0	100.0	100.0	28.6	100.0
Gentamicin	82.1	100.0	80.0	90.0	85.7	100.0
Tobramycin	10.7	16.6	-	41.7	14.3	33.3
Ciprofloxacin	92.9	88.9	90.0	70.0	42.9	100.0
İmipenem	100.0	100.0	80.0	100.0	100.0	100.0
Tetracycline	-	22.2	20.0	20.0	-	16.7
Cotrimoxazole	64.3	55.6	70.0	100.0	14.3	100.0

**Table 2:** Susceptibility rates of Enterobacteriaceae and Acinetobacter baumanii between November 1999 and January 2002 (Microdilution technique).

Antibiotics	Klebsiella spp (n: 86)		A. baumanii (n: 83)		E.coli (n: 30)		Enterobacter spp (n: 24)		Proteus spp. (n: 13)		Serratia spp. (n: 13)	
	
	MIC50/90	Susc. %	MIC50/90	Susc. %	MIC50/90	Susc. %	MIC50/90	Susc. %	MIC50/90	Susc. %	MIC50/90	Susc. %
Ampicillin	8/>16	10.5	16/>16	7.2	8/>16	10.0	8/>16	4.2	8/>16	15.4	8/>16	-
Amoxicillin/clav.	4/>16	77.9	16/>16	7.2	4/>16	13.3	8/>16	4.2	4/>16	84.6	8/>16	-
Aztreonam	2/>16	73.3	8/>16	6.0	2/>16	66.6	2/>16	45.8	2/>16	92.3	2/16	84.6
Piperacillin	16/>64	60.5	16/128	39.7	16/128	30.0	16/128	41.7	16/>64	84.6	16/128	61.5
Ticarcillin	32/>64	25.6	16/128	7.2	16/128	26.7	32/128	54.2	32/>64	53.8	32/128	38.5
Ticarcillin/clav.	16/128	60.5	16/128	9.6	16/128	40.0	16/128	58.3	16/>64	92.3	16/128	61.5
Cefazolin	8/>16	77.9	16/>16	1.2	8/>16	46.7	8/>16	8.3	8/>16	69.2	8/>16	-
Cefuroxime	8/>16	88.4	16/>16	3.6	8/>16	46.7	8/>16	12.5	8/>16	76.9	8/>16	-
Cefotaxime	8/64	96.5	16/64	9.6	16/64	73.3	8/64	58.3	8/64	100.0	8/32	84.6
Ceftazidime	1/>16	84.9	4/>16	42.2	1/>16	66.7	1/32	37.5	1/>16	92.3	1/>16	76.9
Ceftriaxone	16/>32	90.7	16/64	12.0	16/>32	73.3	8/>32	50.0	16/>32	100.0	8/32	84.6
Amikacin	16/>32	93.0	16/>32	54.2	16/>32	93.3	8/>32	100.0	16/>32	100.0	8/>32	92.3
Gentamicin	4/16	89.5	4/16	65.0	4/>8	90.0	4/>8	95.8	4/>8	69.2	4/>8	84.6
Tobramycin	4/16	84.9	4/16	91.5	4/>8	93.3	4/>8	41.7	4/>8	84.6	4/>8	76.9
Ciprofloxacin	1/>2	89.5	1/>2	66.2	1/>2	66.6	1/>2	100.0	1/>2	100.0	1/>2	92.3
İmipenem	2/>8	95.3	4/>8	79.5	2/>8	96.6	2/>8	79.2	2/>8	100.0	2/>8	69.2
Tetracycline	4/>8	81.4	4/>8	56.6	4/>8	63.3	4/>8	79.2	4/>8	15.4	4/>8	15.4
Cotrimoxazole	1/>2	81.4	1/>2	22.9	1/>2	53.3	1/>2	75.0	1/>2	23.1	1/>2	69.2

**Table 3:** Susceptibility (numbers) of imipenem-, gentamicin-, and ciprofloxacin-resistant Acinetobacter baumanii.

	All isolates (n: 90)	Gentamicin-resistant (n: 30)	Imipenem-resistant (n: 18)	Ciprofloxacin-resistant (n: 32)
Ampicillin	6	2	-	-
Amoxicillin/clavulanate	6	3	-	-
Aztreonam	5	1	1	-
Piperacillin	33	4	1	9
Ticarcillin	6	2	-	1
Ticarcillin/clavulanate	8	5	-	1
Cefazolin	2	1	-	-
Cefuroxime	3	-	1	-
Cefotaxime	8	1	1	-
Ceftazidime	36	5	6	7
Ceftriaxone	10	1	1	-
Amikacin	47	2	6	13
Gentamicin	60	-	11	25
Tobramycin	58	24	17	28
Ciprofloxacin	58	21	11	-
İmipenem	72	25	-	26
Tetracycline	47	17	10	6
Cotrimoxazole	20	10	5	6
